# COLLABORATIVE INTERNATIONAL EXPERIENTIAL LEARNING IN GERONTOLOGY: AGING GLOBALLY INITIATIVE

**DOI:** 10.1093/geroni/igae098.0049

**Published:** 2024-12-31

**Authors:** Aleksandra Zecevic, Anne-Marie Boström

**Affiliations:** University of Western Ontario, London, Ontario, Canada; Karolinska Institutet, Stockholm, Stockholms Lan, Sweden

## Abstract

Population aging is greatly impacting future generations of healthcare providers tasked with improving the healthcare systems they are inheriting. In this presentation, we introduce an innovative example of how students, researchers, educators and community partners from Canada and Scandinavia came together to comparatively explore models of health, social care, and welfare to advance global health. Collaborative international experiential learning in gerontology is an approach that broadens the application of academic theory outside classroom in an international context. It includes authentic reflection, develops transferable skills and strengthens employability. Our network supports future generations of healthcare professionals in becoming global-ready graduates. We highlight a collaborative course called Aging Globally: Lessons from Scandinavia, initiated at Western University, Canada in 2018, and delivered in partnership with OsloMet University (Norway), Karolinska Institutet (Sweden) and seven non-academic partners, such as Socialstyrelsen and Silviahemmet (Sweden) and Cycling without Age (Denmark). Over the past seven years, the course involved 425 students and 24 professors from Health Studies, Occupational Therapy, Physiotherapy, Nursing, and Technology, Science and Design programs. Student outcomes include expended knowledge, cultural competencies, and transferable skills. The course was a catalyst for three curriculum development grants totaling CAD $2 million, enrichment of the curriculum with 57 international internships and 13 exchanges, 12 summer courses, and new research partnerships, lifting international education in gerontology to a new level. Presenters will share experiences, evidence of impact on students, and describe joint efforts to inspire social change and sustainability of improved quality of life and well-being for older adults everywhere.

